# Omega-3 Long-Chain Polyunsaturated Fatty Acids Intake in Children with Attention Deficit and Hyperactivity Disorder

**DOI:** 10.3390/brainsci9050120

**Published:** 2019-05-23

**Authors:** Milagros Fuentes-Albero, María Isabel Martínez-Martínez, Omar Cauli

**Affiliations:** 1 Children’s Mental Health Center, Hospital Arnau de Villanova, 46015 Valencia, Spain; milagrosfuentesalbero@yahoo.es; 2 Department of Medicine and Nursing, University of Valencia, 46010 Valencia, Spain; m.isabel.martinez@uv.es

**Keywords:** fish intake, omega-3 fatty acids, nutrients, ADHD, children, diet-deficient

## Abstract

Omega-3 long-chain polyunsaturated fatty acids (LC-PUFA) play a central role in neuronal growth and in the development of the human brain, and a deficiency of these substances has been reported in children with attention deficit hyperactive disorder (ADHD). In this regard, supplementation with omega-3 polyunsaturated fatty acids is used as adjuvant therapy in ADHD. Seafood, particularly fish, and some types of nuts are the main dietary sources of such fatty acids in the Spanish diet. In order to assess the effect of the intake of common foods containing high amounts of omega-3 polyunsaturated fatty acids, a food frequency questionnaire was administered to parents of children with ADHD (*N* = 48) and to parents of normally developing children (control group) (*N* = 87), and the intake of dietary omega-3 LC-PUFA, such as eicosapentaenoic acid (EPA) and docosahexaenoic acid (DHA), was estimated. Children with ADHD consumed fatty fish, lean fish, mollusks, crustaceans, and chicken eggs significantly less often (*p* < 0.05) than children in the control group. The estimated daily omega-3 LC-PUFA intake (EPA + DHA) was significantly below that recommended by the public health agencies in both groups, and was significantly lower in children with ADHD (*p* < 0.05, Cohen’s d = 0.45) compared to normally developing children. Dietary intervention to increase the consumption of fish and seafood is strongly advised and it is especially warranted in children with ADHD, since it could contribute to improve the symptoms of ADHD.

## 1. Introduction

There is a growing evidence that several mental disorders, although they show an underlying genetic predisposition [1], are probably the product of an interplay between genetic susceptibility and environmental factors [2], of which inadequate nutrition may be a component [3,4]. Among the nutrients that have been consistently shown to be related to mental health and to different psychiatric disorders, mention must be made of omega-3 long-chain polyunsaturated fatty acids (LC-PUFA) [5,6,7]. A proper physical and mental health and neurodevelopment require a balanced ratio of omega-3 to omega-6 polyunsaturated fatty acids, but the typical diet in many countries provides a much larger intake of food containing omega-6 as compared to omega-3 LC-PUFA, thus often resulting in an imbalance and deficient omega-3 intake [5,8]. The consumption of supplements containing omega-3 LC-PUFA has been shown to be an effective measure in addition to the administration of psychotropic drugs for treating several psychiatric diseases [9,10,11,12]. In this regard, it has been demonstrated that omega-3 LC-PUFA such as eicosapentaenoic acid (EPA) and docosahexaenoic acid (DHA) may be helpful in the treatment of attention deficit hyperactive disorder (ADHD) in children [13,14,15,16,17]. Whether the pathophysiology of ADHD may be linked to inadequate bioavailability of omega-3 LC-PUFA, and whether it may be counteracted by dietary supplementation or increased intake of foods containing large amounts of omega-3 LC-PUFA, has gained growing interest in part due to the increasing knowledge of the role of nutrition in psychiatric disorders and in ADHD [18,19,20]. Dietary guidelines recommend regular fish consumption in all age ranges as the main source of omega-3 LC-PUFA intake [21].

Previous studies refer to the fundamental role afforded by omega-3 LC-PUFA in several essential metabolic functions, given their implication in diverse neuronal processes, as well as in cell growth, the function of cell membrane, hormonal, and immunological cross-talk, and gene expression regulation [8,22,23]. Alteration of some of these functions has been implicated in the physiopathology of ADHD [24]. Several experimental studies suggest that deficiencies of omega-3 LC-PUFA strongly alter brain function, not only during the developmental stages, but also throughout life [25]. There is some evidence to suggest that omega-3 LC-PUFA homeostasis may be impaired in patients with ADHD as a result of deficits and/or imbalances in nutritional intake, genetic alteration, changes in the activity of the enzymes involved in their metabolism, or the influence of some environmental agents [24,25].

Although many studies on omega-3 LC-PUFA supplementation in ADHD have been published in recent years [13,14], most refer to either interventions performed in patients who were given omega-3 LC-PUFA supplements apart from their normal diets. Remarkably, there are few studies on the intake of omega-3 LC-PUFA through diet in patients with ADHD. The present study was therefore designed with the following three main objectives:

1) Evaluation of the pattern of consumption of the main dietary sources of food containing omega-3 LC-PUFA in children with ADHD and in a control group.
2) Estimation of the daily intake of omega-3 LC-PUFA (EPA + DHA) in the two groups.
3) Evaluation of the influence of age, sex, and body mass index (BMI) upon omega-3 LC-PUFA intake.

## 2. Materials and Methods

### 2.1. Study Design

An observational case-control study was carried out in Valencia (Spain) in 2016–2017. The study participants were recruited among patients (children and adolescents) with ADHD undergoing child psychiatrist consultation. Neurologically healthy children (control group) were recruited from two public schools in Valencia (Spain). Attention deficit hyperactive disorder was confirmed based on the DSM-IV diagnostic criteria using a standard neurodevelopment examination and interview (Conners scale). The parents of children with ADHD were interviewed during ordinary consultation with the child psychiatrist. Clinical information (diagnosis of ADHD, medication, presence of other comorbidities, anthropometric data) was retrieved by reviewing the medical records in the psychiatrist consultation of children with ADHD. Body mass index was calculated as weight in kilograms divided by the square of height in meters. For children and adolescents, BMI is age- and sex-specific, and is often referred to as BMI-for-age. According to the international guidelines, BMI was grouped into four categories: underweight (BMI less than the 5th percentile), normal or healthy weight (5th percentile to less than the 85th percentile), overweight (85th percentile to less than the 95th percentile), or obese (equal to or greater than the 95th percentile) [26].The children in the control group were sex- and age-matched (proportion 1:2) with the children in the ADHD group. Matching increases the efficiency of the estimates if the matching variables are associated with both the disease and exposure. The study comprised 135 children: 48 with a diagnosis of ADHD (age 5–14 years) and 87 with no ADHD or other psychiatric or neurological disorders (age 4–13 years). Socio-economic variables were measured through three variables: First, occupational social class, widely used in Spain as a measure of socioeconomic position [27]; it was defined using a Spanish adaptation of the British social class classification. In this study, we recoded the social status in three categories: higher, medium and lower. Educational level was recorded as primary or less, secondary, or university. Employment situation was categorized as employed, unemployed, and homemaker.

The study protocol was approved by the local Ethics Committee of the University of Valencia (Valencia, Spain) (protocol number H1397475950160). Parents signed the informed consent in order to participate in the study.

### 2.2. Diet Assessment

The parents completed the food frequency questionnaire (FFQ) about their children’s diet and were also instructed to report all beverage and supplement consumption. The instrument was a semi-quantitative food questionnaire that was comprised of 136 food items, and is validated in Spain [28]. Specifically, the parents were instructed to record estimated portion sizes for each item ingested according to a previously validated [29] visual guide to improve the accuracy of their estimates. Consumptions were assessed by crossing the frequency and the portion size for each food. All food records were analyzed using Nutrition Data Systems-Research free software (DIAL®). Nutrient intake was averaged across the three days and normalized to intake per 1000 kcal, to generate the measures used in subsequent analyses. Energy and nutrient intake was calculated from the Spanish food composition tables [30,31].

### 2.3. Estimation of Omega-3 LC-PUFA Intake from Fish and Nuts

Parents self-reported fish and nuts consumption in their children. Fish was defined as “any kind of fish, including fish sticks and canned tuna fish, shellfish, crustaceans and mollusks.” Participants reported: (a) how often they consumed fish (“did not eat,” “once–three times a month,” “about once a week,” “twice–four times a week,” “five–six times a week,” “once a day,” “twice–three time a day”); and (b) the type of fish they typically consumed.

The items of the three-day semi-quantitative food questionnaire [30] related to fish and seafood consumption and their omega-3 LC-PUFA contents (g/100 g of food item, as the sum of EPA + DHA) were: (a) lean fish: young hake, hake, sea bream, grouper, and sole (0.62); (b) fatty fish: salmon, mackerel, tuna, Atlantic bonito, and sardine (1.87); (c) cod (0.70); (d) smoked and salted fish: salmon and herring (4.44); (e) shellfish: mussel, oyster, and clam (2.20); (f) seafood: shrimp, prawn, and crayfish (0.90), and (g) mollusks: octopus, cuttlefish, and squid (0.71). Omega-3 LC-PUFA intake was calculated as frequency × (EPA + DHA) content for each food item (fish, seafood). We also included common foods in Spanish diets containing high amounts of omega-3 LC-PUFA such as dry fruit nuts: walnuts, hazelnuts, and almonds (6.33) [28,32]. We estimated the intake of EPA + DHA because these fatty acids are administered as nutritional supplements in clinical settings for children/adolescents with ADHD. In addition, we asked the parents about the frequency of consumption of omega-3 LC-PUFA supplements or omega-3 fatty acid-enriched milks. The intake of omega-3 LC-PUFA and fish consumption were adjusted for total energy intake using the residuals method proposed by Willett et al. [33].

### 2.4. Statistical Analysis

In the univariate analysis, variables were represented as absolute frequencies and percentages for categorical variables, and as the mean ± standard deviation (SD) for continuous (quantitative) variables. In the bivariate analysis, we first checked for normal or non-normal data distribution for quantitative variables using the Shapiro–Wilk (*n* < 50) or Kolmogorov–Smirnoff (*n* ≥ 50) tests. As a result of non-normal data distribution, we used nonparametric tests, e.g., the Mann–Whitney U-test (when comparing quantitative variables between two groups) or the Kruskal–Wallis test (when comparing quantitative variables among three or more groups). Correlation analysis between quantitative variables was performed with the nonparametric Spearman test. In order to control the effect of intervening variables, partial correlations were performed. Differences between categorical variables were evaluated with the chi-squared test. In the case of food frequencies, we applied the z-test for differences between proportions to determine which of the five to seven categories differed between the control and ADHD groups. To quantify the effect size for two groups comparison we calculated Cohen’s d. Statistical significance was considered to be *p* < 0.05. The SPSS version 24.0 statistical package (SPSS, Inc., Chicago, IL, USA) was used throughout.

## 3. Results

### 3.1. Description of the Sample

The characteristics of the study sample are shown in Table 1. Since ADHD shows a clear male predominance over females of about 3:1 to 4:1 in community-based samples of young individuals [1,2,34], we attempted to mimic the difference in sex distribution in our study: females in the ADHD group represented 25.0%, versus 28.7% in the control group. There were no significant differences between the groups regarding sex distribution (*p* = 0.64) or mean age (*p* = 0.86). Regarding the weight distribution of the subjects, 18.5% (*n* = 25) of the sample had low weight (percentile < 5), 36.3% (*n* = 49) showed normal weight (percentile 5–84), 23.7% (*n* = 32) were overweight (percentile 85–94), and 21.5% (*n* = 29) were obese (≥95 percentile). Significant differences in weight distribution were observed between the control and ADHD groups (*p* < 0.0001). In relation to BMI, low weight was significantly more prevalent in the control group compared to the ADHD group (*p* < 0.0001), while obesity was significantly more frequent in the ADHD group compared to the control group (*p* < 0.001) (Table 1).

### 3.2. Energy Intake and Frequency of Seafood Consumption

**Figure 1:**
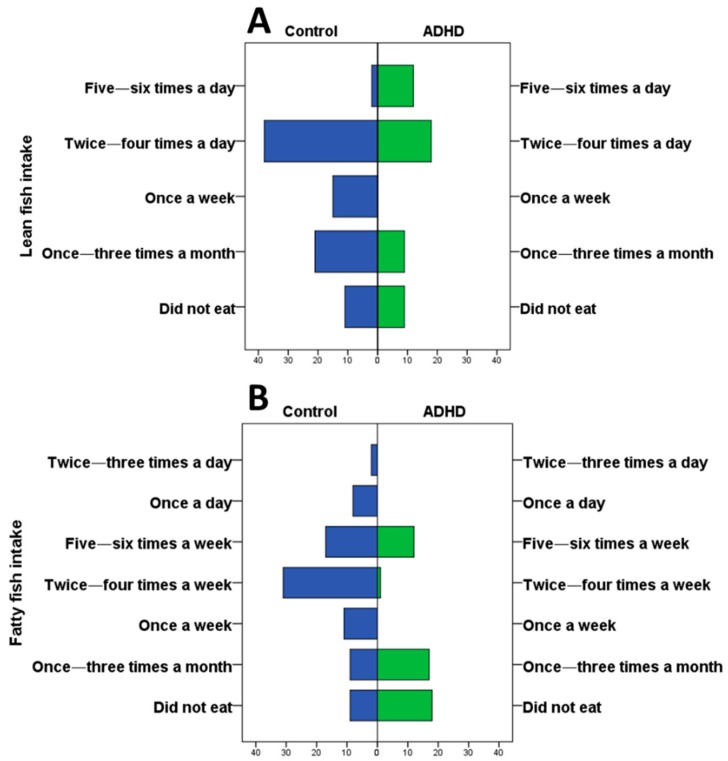
Frequency of intake of lean and fatty fish (Supplementary Tables S1 and S2 for raw).

The reported average energy intake was approximately 1705 kcal. Of this amount, 51% corresponded to carbohydrates, 34% to fat, and 15% to protein. Fish intake was significantly lower in children/adolescents with ADHD than among the controls for all types of fish and seafood, except codfish. Significant differences were recorded in relation to lean fish (including young hake, hake, blackspot sea bream, goliath grouper, and common sole) (*p* < 0.001) (Figure 1A); the z-scores analysis showed significant differences for the intake categories “once a week” (*z*-score = 3.05; *p* < 0.01, higher in the control group), “twice–four times a week” (*z*-score = 2.15; *p* < 0.05, higher in the control group) and “five–six times a week” (*z*-score = −4.14; *p* < 0.001, higher in the ADHD group). Significant differences were also observed in the case of fatty fish (salmon, mackerel, tuna, bonito, sardine) (*p* < 0.001; chi-squared test) (Figure 1B); the *z*-scores analysis showed significant differences for the intake categories “did not eat” (*z*-score = −3.77; *p* < 0.001, higher in the ADHD group), “once–three times a month” (*z*-score = −3.54; *p* < 0.001, higher in the ADHD group), “once a week” (*z*-score = 2.55; *p* < 0.01, higher in the control group), and “twice–four times a week” (*z*-score = 4.39; *p* < 0.001, higher in the control group).

Significant differences were recorded in the intake of smoked fish (including smoked and salted fish such as salmon and herring) (*p* < 0.001; chi-squared test); the z-scores analysis showed significant differences for the intake category “once a week” (*z*-score = 2.17; *p* < 0.05, higher in the control group). The same applied to the intake of shellfish (including mussel, oyster, and clam) (*p* < 0.05); the *z*-scores analysis showed significant differences for the intake category “twice–four times a week” (*z*-score = 2.02; *p* < 0.05, higher in the control group). Likewise, significant differences were observed in the intake of mollusks (including octopus, common cuttlefish, and squid) (*p* < 0.001); the *z*-scores analysis showed significant differences for the intake category “once a week” (*z*-score = 2.82; *p* < 0.01, higher in the control group). Lastly, significant differences were recorded in the intake of crustaceans (including shrimps, prawn, and crayfish) (*p* < 0.01; chi-squared test); the *z*-scores analysis showed significant differences for the intake categories “once a week” (*z*-score = 2.02; *p* < 0.05, higher in the control group) and “five–six times a week” (*z*-score = −2.11; *p* < 0.05, higher in the ADHD group). In contrast, the intake of codfish was not significantly different between the two groups (*p* = 0.23).

There were no significant differences in relation to the consumption of nuts (referred to those containing higher amounts of omega-3 LC-PUFA, such as walnuts and almonds) (*p* = 0.07), omega-3 LC-PUFA supplements (*p* = 0.26), or omega-3 fatty acid-enriched milk (*p* = 0.14). The intake of omega-3 fatty acids from nuts were not included in the calculation of daily EPA + DHA intake, since these foods contain other omega-3 LC-PUFA different from DHA and EPA, and because no significant differences in the intake of dry fruits were observed between the ADHD and control groups.

Significant differences in food intake were observed between females and males in relation to fatty fish and shellfish (being higher in males compared to females, *p* < 0.05), and eggs (again being higher in males compared to females, *p* < 0.01), but not to other foods (*p* > 0.05).

### 3.3. Estimation of Omega-3 LC-PUFA (EPA + DHA) Intake

**Figure 2:**
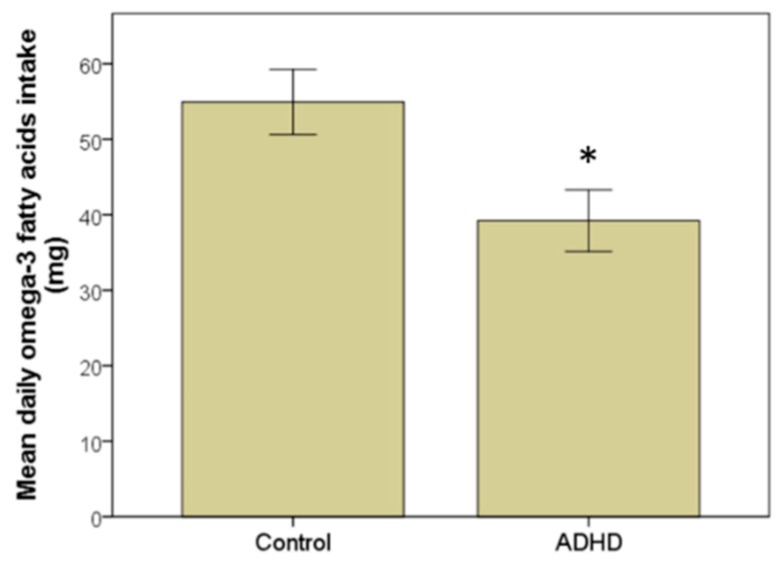
Estimated daily EPA + DHA intake from seafood. Comparison of EPA + DHA intake in the control and ADHD groups. Significant difference reported with an asterisk *, *p* < 0.05.

The estimated ingestion of omega-3 LC-PUFA (EPA + DHA) in the diet was 109.87 ± 80.27 mg/day for the control group and 78.42 ± 56.64 mg/day for the children with ADHD (*p* < 0.01, effect size Cohen’s d = 0.45) (Figure 2). The analysis of the mean intake per day of omega-3 LC-PUFA for each type of fish and seafood is shown in Table 2. There is a significant effects in omega-3 LC-PUFA between the two groups for lean fish (*p* < 0.05), fatty fish (*p* < 0.01), mollusks (*p* < 0.05), and other types of fish and seafood less frequently consumed (*p* < 0.05).

There was a significant correlation between the mean daily intake of EPA + DHA and the frequency of intake of fatty fish (rho = 0.18, *p* < 0.05) and crustaceans (rho = 0.17, *p* < 0.05). No significant differences were observed in the estimated daily amounts of omega-3 LC-PUFA (EPA + DHA) between sexes (*p* = 0.17) or among children in the different weight categories (*p* = 0.57).

In contrast, a significant and direct correlation was observed between the intake of omega-3 LC-PUFA and of the age of the children (rho = 0.21, *p* < 0.05; Spearman test). The correlation between mean daily omega-3 LC-PUFA intake and age no longer proved significant (*p* > 0.05, partial correlation) after controlling for the intervening variables, e.g., group, sex, and weight categories, suggesting that these contribute significantly to the association between omega-3 LC-PUFA intake and age.

## 4. Discussion

Nowadays, several studies showing that food is not only useful for providing energy for bodily functions [35], but it can also prevent or moderate several diseases and a proper diet can improve both physical and mental health [4,5,25,36,37,38]. Omega-3 LC-PUFA supplementation has been shown to produce beneficial effects in children with ADHD, as summarized by two recent meta-analyses, although some conflicting results have been also reported [12,13,14,15]. To our knowledge, no studies have explored whether the intake of the omega-3 LC-PUFA EPA and DHA (expressed as mg/day) through the diet is adequate in children with ADHD. The European Food Safety Authority (EFSA) recommends an average EPA + DHA intake of 250 mg/day in the pediatric population [21]. The Food and Agriculture Organization (FAO)/World Health Organization (WHO) [39] recommends an intake of EPA + DHA about 100–200 mg/day for children aged 2–6 years and 200–250 mg/day from age 6 years onwards. Our study shows worrying results in the form of a low intake of EPA + DHA in both the control group and the ADHD group compared to the amount recommended by the public health organizations (50%–60% reduction with respect to the recommended daily dose) [21,39,40,41]. Similar findings have also emerged from a recent French population-based study in children (3–10 years) and adolescents (11–17 years) [42]. The mean daily intake of EPA + DHA correlated significantly to age, though on correcting for BMI, which also increases with age, we still observed a low intake of these essential molecules. Interestingly, a lower intake of omega-3 LC-PUFA has also been recently reported in children with autism spectrum disorder [43] suggesting it may be a general nutritional problem affecting the pediatric population rather than a problem conditioned by some specific neuropsychiatric disorder. However, it must be pointed out that the consequences of an omega-3 LC-PUFA (EPA + DHA) deficient diet may have even worse deleterious effects in children with neurodevelopmental disorders, taking into account that omega-3 LC-PUFA supplementation has been shown to afford beneficial effects when added to the pharmacological treatment of ADHD [13,14,15,38,40]. The analysis and the evolution of ADHD symptoms in children with low versus normal omega-3 LC-PUFA intake deserves future investigations in order to assess its role in ADHD symptomatology. Besides omega-3 LC-PUFA, reduced fish intake could lead to other nutrients deficiencies, such as phospholipids, the neuromodulator amino acid taurine, high-quality source of protein, and beneficial marine carotenoids such as astaxanthin [44], which have been demonstrated to possess anti-oxidant properties and anti-inflammatory effects [44,45,46,47,48], and regular fish intake reduces hyperlipidemia [49], which in turn can improve brain function [50]. 

Another emerging nutritional concern in our study was the high prevalence of obesity in children with ADHD (40% of the sample compared to 12% of the control group). This finding agrees with those of a recent meta-analysis concluding that the prevalence of obesity in ADHD is 40% higher than in the general population [51,52]. The causes of being overweight and child obesity are multifactorial (diet, sedentary lifestyle, socioeconomic status, disease conditions, neurodevelopmental disorders, etc.), but the core symptoms of children with ADHD might contribute to such increased rates [43,51,52]. Among these factors, ADHD symptoms, such as inattention or impulsivity, can increase the risk of obesity by increasing and dysregulating the food intake pattern in several ways (excessive eating, binge eating, unhealthy food choices, etc.) [51]. Attention deficit disorder may be associated with not remembering whether eating has been done or with a lack of satiation feeling [51,52]. Given the lack of planning and self-regulation skills, the patient can lose control over food and reduce the time spent doing physical exercise [40]. Also, impulsivity may contribute to excessive food intake in ADHD, even in the form of binge eating. This anomalous eating pattern could produce a net increase in adipose tissue, which affects the severity of ADHD and vice versa. Bowling et al. [43] concluded that more ADHD symptoms predict higher fat mass at later ages, which further confirms that more symptoms of impulsivity may contribute to being overweight. Longitudinal studies have explored the direction of the link between ADHD and obesity. Some studies suggested that ADHD precedes, and likely contributes to, subsequent overweightness and obesity [51,52]; however, the reverse pattern has also been demonstrated in preschool children [53]. One of the proposed pathophysiological mechanism by which being overweight may contribute to ADHD relates to sleep-disordered breathing [54], leading to an excessive daytime sleepiness, which in turn may promote inattention via hypoxemia, which in turn contributes to altered prefrontal functioning [52,54]. Finally, a common genetic mechanism between ADHD and obesity has been also proposed [55]. Although the mechanism underlying the association is still unknown, preliminary evidence suggests the role of the dopaminergic reward system [56] or melanocortin system [57]. It is indeed possible that bidirectional pathways are likely involved.

Our study has a number of limitations. First, the cross-sectional observational design involved limits regarding inferences about causality between insufficient intake of omega-3 LC-PUFA and the worsening of ADHD symptoms. Second, there were a number of issues related to the completion of records. Data referred to intake may contain errors due to inaccuracies in recorded quantities and they are based on parents’ reports rather than children’s. However, we are confident that the self-reported information provided by parents about the nutritional assessment of their children was adequate because they showed interest in the study and they received training and support in filling out the food records. Furthermore, the attrition rate was low. We, therefore, think that the study has a good internal validity. A third limitation is the fact that we did not measure the intake of omega-3 LC-PUFA coming from other sources. Nevertheless, we are confident about the main role of fish and seafood as the principal source of EPA + DHA in the Spanish diet [36,39].

In our sample of children with ADHD and controls (age- and sex-matched with the ADHD subjects), there were considerably more boys than girls (reflecting the characteristic sex ratio observed in ADHD [1,2]), which can rule out a proper analysis for the effects of sex. Both the controls and the ADHD children were recruited not only from the same age group but also from the same geographical region, and had a similar socioeconomic status. Data were collected over the same time period (winter), and this homogeneity reduced potential sources of bias. 

Despite these limitations, our study underscores the need for greater attention to the education of parents and children regarding healthy dietary habits in Spain, and as such, education is the most promising and practical complementary management strategy in ADHD. Given that fish consumption is the main source of dietary omega-3 LC-PUFA [58], interventions promoting fish consumption in a balanced diet, as well as other positive eating behaviors, are strongly warranted in the future.

## 5. Conclusions

The intake of seafood in particular fish, is reduced in children with ADHD compared to typically developing children and this may contribute to reduced intake of some omega-3 LC-PUFA such as EPA and DHA, essential nutrients for a proper brain development and function. Further research is required to clarify associations between ADHD symptomatology, eating patterns and health status.

## Figures and Tables

**Table 1:** Characteristics of the study sample.

Variable	Control	ADHD	*p*-Value
Age	10.00 ± 0.27 (range 4–13)	9.54 ± 0.31 (5–14)	*p* = 0.86 (Mann–Whitney test)
Sex	Female *n* = 25 Male *n* = 62	Female *n* = 12 Male *n* = 36	*p* = 0.64 (Chi-squared test)
BMI	18.69 ± 0.39 (range 10.65–30.44)	20.89 ± 0.44 (range 15.50–28.31)	*p* = 0.04 (Mann–Whitney test)
Low weight	26.4%	4.2%	*p* < 0.001 (Chi-squared test)
Normal weight	36.8%	35.4%	
Over weight	25.3%	20.8%	
Obesity	11.5%	39.6%	
Social class	Higher: 26.4%	Higher: 31.3%	*p* = 0.88 (Chi-squared test)
	Medium: 55.2%	Medium: 52.1%	
	Lower: 18.4	Lower: 16.6%	
Employment situation	Father Employed: 97.7% Unemployed: 2.3%	Father Employed: 95.8% Unemployed: 4.2%	*p* = 0.95 (Chi-squared test)
	Mother Employed: 50.6% Unemployed: 16.1% Homemaker: 33.3%	Mother Employed: 58.3% Unemployed: 10.4% Homemaker: 31.3%	*p* = 0.78 (Chi-squared test)
Educational level	Father Primary school: 23.0% Secondary school: 54% University: 23.0%	Father Primary school: 20.8% Secondary school: 54.2 University: 25.0%	*p* = 0.89 (Chi-squared test)
	Mother Primary school: 17.2% Secondary school: 56.4% University: 26.4%	Mother Primary school: 12.5% Secondary school: 56.2% University: 31.3%	*p* = 0.84 (Chi-squared test)

No significant differences in the socio-economic variables were observed between parents in the ADHD and control group such as social class, employment situation, and educational level (Table 1).

**Table 2:** Estimation of Omega-3 LC-PUFA intake form different type of fish and seafood.

Group	Lean Fish (mg/day)	Fatty Fish (mg/day)	Mollusks (mg/day)	Crustaceans (mg/day)	Other Types (mg/day)
Control	45.56 ± 19.81	40,63 ± 33.6	18.28 ± 18.20	3.21 ± 6.22	2.20 ± 5.11
ADHD	38.51 ± 19.22 *	26.42 ± 20.30 **	10.21 ± 15.4 *	3.0 ± 6.43	0.29 ± 2.62 *

*, *p* < 0.05; **, *p* < 0.01 compared to the control group. On considering the daily intake related to weight category, a significant difference was seen to persist between the control and ADHD groups (*p* < 0.01).

## Supplementary Materials

The following are available online at https://www.mdpi.com/2076-3425/9/5/120/s1, Table S1: Frequency of lean fish intake, Table S2: Frequencies of fatty fish intake.
