# Supplementary material for: Omega-3 Long-Chain Polyunsaturated Fatty Acids Intake in Children with Attention Deficit and Hyperactivity Disorder
Source: Brain Sci. 2019 May 23;9(5):120. doi: 10.3390/brainsci9050120 (PMC6562756; doi:10.3390/brainsci9050120)
Supplement: Supplementary file 1 [file brainsci-09-00120-s001.pdf]

Table S1: Frequency of lean fish intake

		GRUPO		
		Control	ADHD	Total
Lean fish	Did not eat	11	9	20
	Once-three times a month	21	9	30
	Once a week	15	0	15
	Twice-four times a week	38	18	56
	five-six times a week	2	12	14
Total		87	48	135

Table S2: Frequencies of fatty fish intake

		GRUPO		
		Control	ADHD	Total
Fatty fish	Did not eat	9	18	27
	Once-three times a month	9	17	26
	Once a week	11	0	11
	Twice-four times a week	31	1	32
	Five-six times a week	17	12	29
	Once a day	8	0	8
	Twice-three time a day	2	0	2
Total		87	48	135
